# The effect of bipolar bihemispheric tDCS on executive function and working memory abilities

**DOI:** 10.3389/fpsyg.2023.1275878

**Published:** 2024-01-03

**Authors:** Adam J. Toth, Cliodhna Harvey, Hannah Gullane, Niall Kelly, Adam Bruton, Mark J. Campbell

**Affiliations:** ^1^Department of Physical Education and Sport Sciences, Faculty of Education and Health Sciences, University of Limerick, Limerick, Ireland; ^2^Lero Institute, University of Limerick, Limerick, Ireland; ^3^Department of Life Sciences, Brunel University London, Uxbridge, United Kingdom; ^4^School of Life and Health Sciences, University of Roehampton, London, United Kingdom; ^5^The Science Foundation Ireland Center for Software Research, Lero Institute, University of Limerick, Limerick, Ireland

**Keywords:** transcranial direct cortical stimulation (tDCS), executive functions, working memory (WM), left dorsolateral prefrontal cortex (DLPFC), neuromodulation

## Abstract

**Introduction:**

Cognitive functioning is central to the ability to learn, problem solve, remember, and use information in a rapid and accurate manner and cognitive abilities are fundamental for communication, autonomy, and quality of life. Transcranial electric stimulation (tES) is a very promising tool shown to improve various motor and cognitive functions. When applied as a direct current stimulus (transcranial direct current stimulation; tDCS) over the dorsolateral pre-frontal cortex (DLPFC), this form of neurostimulation has mixed results regarding its ability to slow cognitive deterioration and potentially enhance cognitive functioning, requiring further investigation. This study set out to comprehensively investigate the effect that anodal and cathodal bipolar bihemispheric tDCS have on executive function and working memory abilities.

**Methods:**

72 healthy young adults were recruited, and each participant was randomly allocated to either a control group (CON), a placebo group (SHAM) or one of two neurostimulation groups (Anodal; A-STIM and Cathodal; C-STIM). All participants undertook cognitive tests (Stroop & N Back) before and after a 30-minute stimulation/ sham/ control protocol.

**Results:**

Overall, our results add further evidence that tDCS may not be as efficacious for enhancing cognitive functioning as it has been shown to be for enhancing motor learning when applied over M1. We also provide evidence that the effect of neurostimulation on cognitive functioning may be moderated by sex, with males demonstrating a benefit from both anodal and cathodal stimulation when considering performance on simple attention trial types within the Stroop task.

**Discussion:**

Considering this finding, we propose a new avenue for tDCS research, that the potential that sex may moderate the efficacy of neurostimulation on cognitive functioning.

## Introduction

Cognitive functioning is central to the ability to learn, problem solve, remember, and use information in a rapid and accurate manner (Morley et al., 2015). Fiocco and Yaffe (2010) highlighted that cognitive abilities are fundamental for communication, autonomy, and quality of life. It has been well established that those who experience cognitive impairment show a decreased ability to execute daily living activities, and are at increased risk of mortality, compared to those with no cognitive impairment (Johnson et al., 2007). Two fundamental aspects of cognition are executive functioning (EF) and working memory (WM) (Timmann and Daum, 2007). EF involves the ability to focus attention, plan and attend to task-relevant information in a ‘noisy' environment (Dubreuil-Vall et al., 2019). Borghini et al. (2018) emphasized the importance EF has on cognitive functioning, and explain that a key attribute of EF is the ability to ignore task-irrelevant information and maintain focus of attention. In conjunction with EF, working memory (WM) refers to the system that maintains newly acquired information in the mind for rapid retrieval while performing complex tasks such as reasoning, comprehension and learning (Fregni et al., 2005; Baddeley, 2010; Logie, 2011; Grot et al., 2017; Al Qasem et al., 2022).

To evaluate performance of EF and WM among individuals, two well established tasks administered within the literature are the Stroop Task (Stroop, 1935) and N-Back letter (Kirchner, 1958) task respectively. The Stroop task tests the ability to shift one's attention (Spreen and Strauss, 1998) in the presence of distraction, or, alternatively to suppress irrelevant information and maintain attentional focus. It is believed to provide a measure of cognitive inhibition (Boone et al., 1990; Archibald and Kerns, 1999). Alternatively, the N-Back task, presents participants with a continual stream of stimuli at fixed intervals, and participants must determine whether each stimulus matches the one presented ‘N' items before. An advantage of the test is that processing load can be varied systematically by manipulating the value of N, which alters both accuracy and reaction time (RT) (Jonides et al., 1997).

The importance of WM and EF can be readily observed among individuals suffering deficits in these cognitive abilities. For example, both WM and EF deficits are among the most common symptoms associated with Alzheimer's disease (AD) (Stopford et al., 2012). In addition to AD, deterioration in EF and WM performance has been associated with numerous neurological and mental disorders, including schizophrenia, attention-deficit/hyperactivity disorder (ADHD), major depressive disorder (MDD), bipolar affective disorder, mild cognitive impairment (MCI), post-traumatic stress disorder, traumatic brain injury, epilepsy, and neurodegenerative dementia and movement disorders (Stegmayer et al., 2015; Maehler and Schuchardt, 2016; Grot et al., 2017; Le et al., 2017; Dubreuil-Vall et al., 2019). Finally, aging is associated with deficits in WM which reduce one's ability to process and maintain task-irrelevant information (Pelosi et al., 2000; Gruber et al., 2011; Le et al., 2017). Due to the impact that WM and EF deficits have on independence and quality of life as one ages or experiences disease, significant research attention has been allocated toward improving these cognitive abilities in clinical (Li et al., 2021), aging (Giuli et al., 2016) and in young healthy populations (Schmiedek et al., 2014). One tool that has emerged as a promising candidate for augmenting cognitive abilities in these populations is neurostimulation.

Among a variety of neurostimulation techniques that currently exist, transcranial electric stimulation is a promising tool shown to improve various motor (Abdelmoula et al., 2016; Angius et al., 2016; Saruco et al., 2017; Toth et al., 2019) and cognitive (Antal et al., 2001, 2004; Kwon et al., 2008; Sparing et al., 2009; Fregni et al., 2015) functions. Most commonly applied as a direct current stimulus (transcranial direct current stimulation; tDCS), this form of neurostimulation has been shown to slow cognitive deterioration (Murugaraja et al., 2017) and potentially enhance cognitive functioning (Javadi and Walsh, 2012; Dubreuil-Vall et al., 2019; Figeys et al., 2021), particularly when applied over the dorso-lateral pre-frontal cortex. tDCS is a non-invasive brain stimulation approach which applies a weak current ~1–2mA over a target region of the cortex to affect the excitability of the underlying neurons. Typically, anodal stimulation involves the depolarization of cortical neurons, thus increasing cortical excitability (Kwon et al., 2008). Cathodal stimulation is understood to have the opposite effect, decreasing cortical excitability (Thair et al., 2017). However, this knowledge largely stems from work investigating the impact of tDCS on motor networks. When used to probe regions predominantly involved in cognitive functioning, results are less clear, with some studies finding positive cathodal effects with no anode effects (Jacobson et al., 2012).

Considering the effects of tDCS specifically on EF and WM abilities, limited work exists among young healthy adults, with some concluding that anodal tDCS over the left DLPFC can enhance WM, with no effect of cathodal stimulation (Fregni et al., 2005; Baumert et al., 2020). Alternatively, anodal stimulation of the left posterior parietal lobe has been shown to worsen working memory performance (Talsma et al., 2017). For EF, conclusions are also mixed with some studies claiming improvements in response inhibition (Loftus et al., 2015; Friehs et al., 2021) while others suggest stimulation leads to increased impulsivity (Shen et al., 2016). While many tDCS studies have discussed targeting the left DLPFC, the right DLPFC remains largely unexamined with little evidence that this area might be involved in working memory (Wu et al., 2014). Moreover, most tDCS paradigms have primarily involved monopolar stimulation of the left DLPFC as opposed to bipolar, bihemispheric monatages. In a study by Waters et al. (2017), they demonstrate the role of the ipsilateral hemisphere has in motor tasks and highlight the increased efficacy of bihemispheric compared to unipolar stimulation. This presents an opportunity as little work has examined the effect of bipolar DLPFC tDCS on EF and WM performance to date.

The purpose of this study is to test whether bihemispheric tDCS over the left DLPFC can improve WM and EF abilities in young adults, evaluated using the N-Back letter and Stroop tasks respectively. We first hypothesize that sensitivity on the N-Back task, and response times and accuracy on the Stroop task, will improve between pre and post stimulation attempts for control (no tDCS) and placebo (sham tDCS) groups. Secondly, we hypothesize that those receiving bihemispheric tDCS with the anode placed over the left DLPFC will show performance improvements on N-Back and Stroop tasks over and above those observed for control and sham groups. Finally, we hypothesize that those receiving bihemispheric tDCS with the anode placed over the right DLPFC will show blunted performance improvements between pre and post N-Back and Stroop tasks compared to those observed for control and sham groups.

## Methods

### Participants

A total of 72 healthy young adults [36 female; age 22.97 ± 3.44 years (mean ± SD)] with no neurological disorders provided informed written consent prior to participating in the study. Participants were instructed to refrain from alcohol 24 h prior and caffeine 6 h prior to participation in the study. Each participant was randomly allocated to one of four groups such that nine male and nine female participants were allocated to each group: a control group (CON), a placebo group (SHAM) and two neurostimulation groups (a-STIM and c-STIM; described below). The study was approved by the university research ethics committee in accordance with the declaration of Helsinki.

### Cognitive tasks

Inquisit 5 software (Millisecond Software LLC) was used to administer Stroop and N-Back Letter tasks and collect data regarding participant performance.

### Stroop task

The Stroop task has been extensively adopted for neuropsychological testing (Scarpina and Tagini, 2017). During the task, participants were presented with one of 4 words (“red,” “green,” “black,” or “blue”) or a colored rectangle (in one of the same 4 colors) on a white background. Words were also presented in red, green, black, or blue colored font. Stimuli were categorized into three different trial types. Congruent trials contained words written in the same color font (i.e., “blue” presented in blue font). Incongruent trials contained color words written in a font of a different color (i.e., “blue” presented in green font). Control trials were those containing colored rectangles. Participants responded to a total of 84 trials during the task with seven trials involving each of the four colors within each trial type. Participants were instructed to always respond to the font color and not the word, as accurately and quickly as possible. Participants pressed the keys on the keyboard “d,” “f,” “j,” and “k” which corresponded respectively to the answers red, green, blue and black. The key bindings were represented at the top of the screen in gray ink throughout the duration of the task. Errors and response times (RT; in milliseconds) were recorded for each trial.

### N-Back letter task

The N-Back Letter task used in this study was adapted to include 0-back, 1-back and the 2-back blocks (3-back excluded). During each block of the task, participants were presented with a stream of the following consonants in white font on a black screen, one after the other: B, C, D, F, G, H, J, K, L, M, N, P, Q, R, S, T, V, W, Y, Z. Each letter was presented on the screen for 500 ms, the screen then remained blank for 3000 ms until the next stimulus showed.

During the 0-Back block, the first consonant presented was the target letter and participants had to remember this one letter and indicate every time this letter appeared in the sequence of presented letters by pressing the “A” key on the keyboard. For the 1-Back block, participants were asked to press the “A” key if the current letter presented was the same as the letter shown previously in the sequence. For the 2-Back block participants were asked to press the “A” key if the current letter presented was the same as the letter presented two letters before. The participants completed a short practice sequence of each block once and then completed 3 test sequences of each block presented in order of difficulty (0-Back → 1-Back → 2-Back). We recorded the number of hits (correct recognition of the target letter), correct rejections (correct recognition of a non-target letter), misses (failed recognition of a target letter), and false alarms (indicating falsely that a non-target letter was a target letter).

### Transcranial direct current stimulation

Two identical bespoke neurostimulation devices designed by Flow Neuroscience (Flow™) (https://www.flowneuroscience.com) were used to administer 2mA of bihemispheric tDCS to the DLPFC of participants in the A-STIM and C-STIM groups. Those in the A-STIM group used the device with the anode and cathode over the left and right DLPFC respectively. Alternatively, those in the C-STIM group used the device with the anode and cathode reversed, that is, over the right and left DLPFC respectively. Saline sponges were fixed to two 22.9 cm^2^ spheric electrodes (current density = 0.09 mA/cm^2^) and current was delivered for 30 min. Those participants in the SHAM group wore the same headset as the A-STIM participants, however, the current was only increased to only 1mA and then back to 0mA over two 30s intervals, and then remained off for the remainder of the 30 min intervention. Finally, those participants in the CON group wore the headset but it was never turned on. To maintain a similar cognitive engagement of participants across groups (Toth et al., 2019), all participants during the 30 min played tetris.

### Protocol

Participants began by providing demographic information, including their age, sex, color blindness, and concussion history. Any participants who were color-blind or had had a concussion in the last 5 years were excluded from participating (no participants excluded). Following this, participants completed the Brunel Mood Scale Questionnaire (BRUMS), to assess their current mood state. Following completion of the BRUMS, each participant performed baseline attempts of the Stroop and the N-Back tasks. The order of presentation of the two tasks was randomized for each participant. Following the baseline attempt at both cognitive tests, participants completed the 30-min neurostimulation intervention according to their group allocation (CON, SHAM, A-STIM, C-STIM). After completing the intervention phase of the protocol, participants completed the BRUMS a second time as well as a post test of the Stroop and N-Back Letter tasks in the same order as they did at baseline. Finally, after completing the experiment, each participant indicated whether they believed they had received neurostimulation during the 30-min intervention.

### Data processing

For each trial type of the Stroop task (Control, Congruent, Incongruent) RTs within baseline or post tests were averaged across like trials to provide an average RT for each participant. Errors were counted to calculate the % of trials participants correctly responded to for a given trial type (Percent Correct).

For the N-Back Task, 1-Back and 2-Back Hits, Misses, Correct Rejections and False Alarms were used to calculate sensitivity on these blocks of the task (D-Prime; d'). D-Prime was calculated as the difference between the z transforms of the hit and false alarm rates [d' = z (Hit Rate) – z (FA rate)] (Macmillan and Creelman, 1990). The hit rate was calculated as [hits/(hits + misses)]. Where the hit rate was 1, an adjusted hit rate was calculated (n-0.5)/n, where n refers to the number of target trials (for the task used in this study, the number of target trials across three iterations of a given block was 15). The False Alarm rate was calculated as false alarms/(false alarms + correct negative). Where the false alarm rate was 0, an adjusted false alarm rate was calculated as (0.5/n) where n refers to the number of non-target trials (for the task used in this study, the number of non-target trials across three iterations of a given block was 30).

### Data analysis

Data analyses were performed using SPSS version 28. Normality of data residuals were assessed through observing Shapiro-Wilk statistics and histogram plots and heterogeneity of variance was assessed using a Levene's test. Where variance heterogeneity was violated, Greenhouse-Geisser corrections were applied. Where *post-hoc* comparisons were made, Sidak alpha adjustments were applied.

To assess the hypothesis that sensitivity on the N-Back task, and response times and accuracy on the Stroop task, would improve between pre and post stimulation attempts for control (no tDCS) and placebo (sham tDCS) groups, we performed separate paired samples *t*-tests comparing baseline and post test scores for each group.

To assess the hypothesis that anodal and cathodal stimulation would respectively improve and disimprove Stroop and N-Back task performance compared to those in the control and placebo (sham) conditions, we performed 2-way (Sex by Condition) ANCOVAs, with baseline scores inputted as a covariate in the model.

Finally, we conducted 3-way (Sex by Condition by Session) ANOVAs on each of the 8 mood categories assessed through the BRUMS questionnaire. We note that the inclusion of sex in the above two models was predicated on the ability to recruit equal numbers of males and female participants and effects examined were exploratory.

## Results

### Practice effect

Paired *t*-tests revealed a practice effect for all measures of the Stroop task except Accuracy (percent correct) on Control trials (Table 1). It also revealed a practice effect for performance on the 2-Back, but not 1-Back, block of the N-Back task (Table 1).

**Table 1:** Paired *t*-tests analysis of practice effects for Stroop and N-Back tasks.

**Task**	**Metric**	**Stimulus type**	**Baseline vs. Post (*p*-value)**
Stroop	Accuracy (Percent Correct)	Control	0.544
		Congruent	**0.029**
		Incongruent	**< 0.001**
	Response Time (RT; ms)	Control	**< 0.001**
		Congruent	**< 0.001**
		Incongruent	**< 0.001**
N-Back	Sensitivity (D-Prime; d')	1-Back	0.927
		2-Back	**0.003**

Bold values denote statistical significance *p* < 0.05.

### Neurostimulation effect

**Figure 1:**
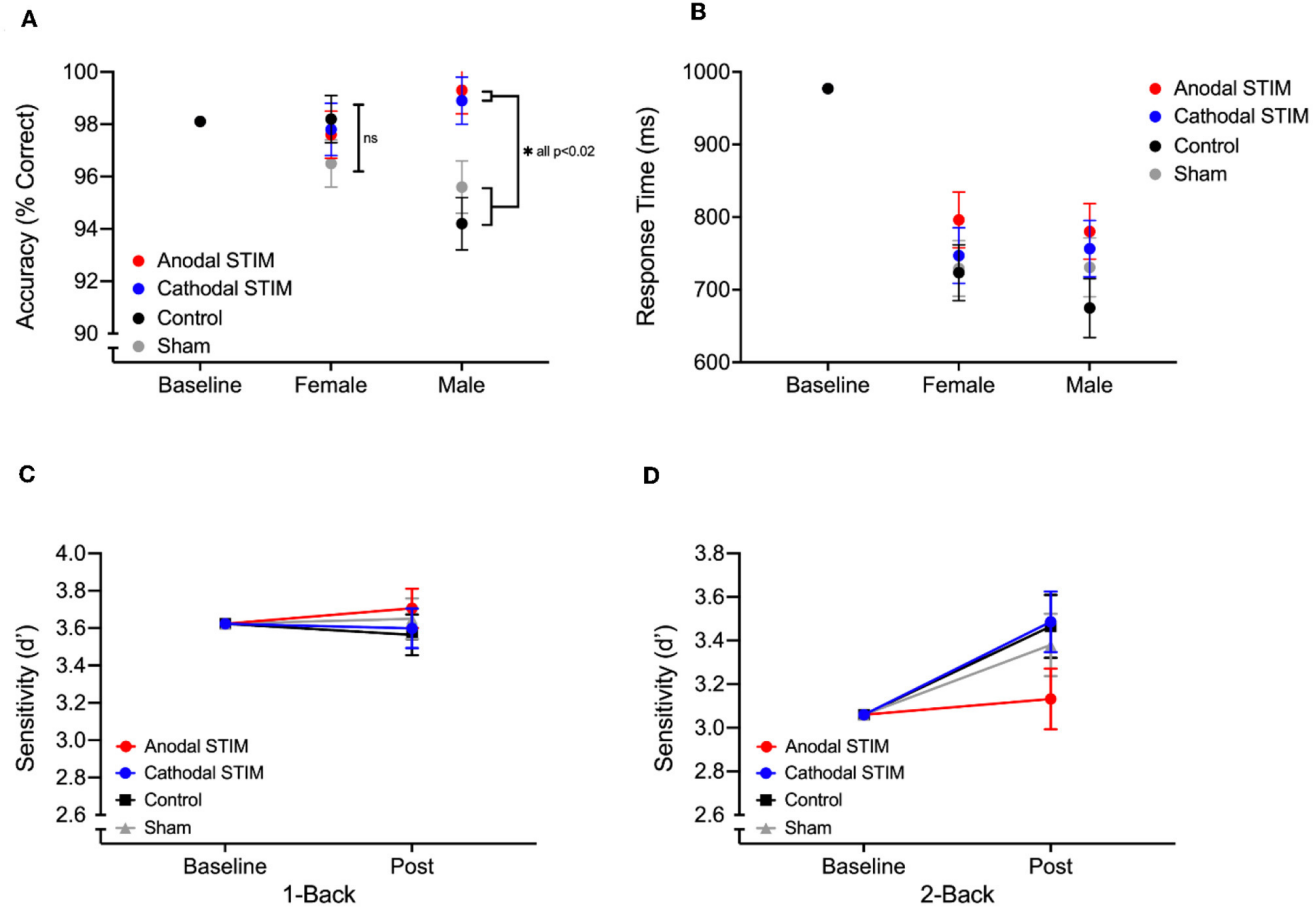
Post test stroop accuracy **(A)** and response times **(B)** on congruent trials of the Stroop task by male and female participants in each of the 4 stimulation groups. ANCOVA adjusted means are displayed with standard errors with covariate baseline score represented with the black circle. **(C, D)** show adjusted means of sensitivity on Post test 1-Back and 2-Back levels of the N-Back task, with covariate baseline scores.

For Stroop task metrics, a significant main effect of Condition was observed for Accuracy on congruent trials [*F*_(3, 69)_ = 3.783, *p* = 0.015, [[Mathtype-mtef1-eqn-1.mtf]]$\eta_{p}^{2}$ = 0.157) as was a significant interaction between sex and condition [*F*_(3, 69)_ =3.628, *p* = 0.018, $\eta_{p}^{2}$ = 0.151]. *Post-hoc* analysis showed that for Male participants, accuracy on post test congruent trials was significantly greater following both anodal and cathodal stimulation when compared to those receiving no stimulation (Control) or a sham stimulation (placebo) (ASTIM-Control *p* < 0.001; ASTIM-Sham *p* = 0.008; CSTIM-Control *p* = 0.001; CSTIM-Sham *p* = 0.02) (see Figure 1). No significant main effect of Sex, Condition or interaction effect was found for any other Stroop task metric (Table 2).

**Table 2:** Statistical results from 2-way ANCOVAs on all metrics.

**Test**	**Metric**	**Stimulus type**	**Effect**	**df1**	**df2**	***F*-value**	***p*-value**	**Effect size**
Stroop	Accuracy (% Correct)	All	Sex	1	69	0.006	0.941	0
			Condition	3	69	1.102	0.355	0.051
			Sex^*^Condition	3	69	1.602	0.198	0.073
		Control	Sex	1	69	0.178	0.674	0.003
			Condition	3	69	0.869	0.462	0.041
			Sex^*^Condition	3	69	0.282	0.838	0.014
		**Congruent**	Sex	1	69	0.514	0.476	0.008
			**Condition**	**3**	**69**	**3.783**	**0.015**	**0.157**
			**Sex** ^*^ **Condition**	**3**	**69**	**3.628**	**0.018**	**0.151**
		Incongruent	Sex	1	69	0.19	0.665	0.003
			Condition	3	69	0.512	0.676	0.025
			Sex^*^Condition	3	69	0.227	0.877	0.011
	Response Time (ms)	All	Sex	1	69	0.851	0.36	0.014
			Condition	3	69	1.858	0.146	0.084
			Sex^*^Condition	3	69	1.265	0.294	0.059
		Control	Sex	1	69	0.108	0.744	0.002
			Condition	3	69	1.458	0.235	0.067
			Sex^*^Condition	3	69	1.159	0.333	0.054
		Congruent	Sex	1	69	0.232	0.632	0.004
			Condition	3	69	1.864	0.145	0.084
			Sex^*^Condition	3	69	0.218	0.884	0.011
		Incongruent	Sex	1	69	2.21	0.142	0.035
			Condition	3	69	1.082	0.364	0.051
			Sex^*^Condition	3	69	1.91	0.137	0.086
N-Back	D-Prime (d')	1-Back	Sex	1	69	0.315	0.577	0.005
			Condition	3	69	0.334	0.801	0.016
			Sex^*^Condition	3	69	0.461	0.71	0.022
		2-Back	Sex	1	69	1.685	0.199	0.027
			Condition	3	69	1.355	0.265	0.062
			Sex^*^Condition	3	69	0.112	0.953	0.005

Bold values denote statistical significance *p* < 0.05.

For N-Back task metrics, no significant main effect of sex, Condition or interaction effect was found for either 1-Back or 2-Back performance (Table 2). A trend was observed however, suggesting post test performance improvements on the 2-Back were blunted by the anodal stimulation (Figure 1).

When observing results from each of the 3-way ANOVAs on the 8 moods captured by the BRUMS questionnaire, we noticed a significant effect of Time for Tension [*F*_(1, 62)_ =22.882, *p* < 0.001, $\eta_{p}^{2}$ = 0.27], Confusion [*F*_(1, 62)_ =5.489, *p* = 0.022, $\eta_{p}^{2}$ = 0.081] and calmness [*F*_(1, 62)_ = 8.272, *p* = 0.006, $\eta_{p}^{2}$ = 0.118], demonstrating all participants overall were less tense, confused, and calm at post test compared to baseline. We also observed a main effect of condition for vigor [*F*_(1, 62)_ =5.489, *p* = 0.022, $\eta_{p}^{2}$ = 0.081], happiness [*F*_(1, 62)_ =5.489, *p* = 0.022, $\eta_{p}^{2}$ = 0.081] and calmness [*F*_(1, 62)_ =5.489, *p* = 0.022, $\eta_{p}^{2}$ = 0.081], demonstrating participants in the CSTIM group overall had lower vigor, happiness and calmness compared to those in any other group. Finally, an interaction between time and condition was observed for vigor [*F*_(3, 62)_ =3.332, *p* = 0.025, $\eta_{p}^{2}$ = 0.139], such that an effect of time was observed for only those in the ASTIM group. Specifically, baseline vigor was significantly higher than post test vigor for the ASTIM group only. See Appendix 1 for a statistical summary of BRUMS data.

## Discussion

This study set out to examine the effect of anodal and cathodal tDCS over the DLPFC on executive functioning and working memory abilities, as evaluated using the color-word Stroop and N-Back letter tasks respectively. In line with our first hypothesis, we found performance on both the Stroop and N-Back tasks to significantly improve between baseline and post-tests, confirming the existence of a practice effect for both cognitive tasks. We then evaluated whether anodal and/or cathodal stimulation modulated post test performance on either task compared to sham and control groups. We found that for the Stroop task, both anodal and cathodal stimulation significantly improved accuracy at post test compared to sham and control conditions only for male participants, with no significant difference observed for response time. On the N-Back task, improvements were observed for 2-Back sensitivity in all groups except those in the A-STIM group, where any practice effect on the 2-Back level of the N-Back task appeared blunted. We discuss the relevance of these findings considering the existing work to date investigating the effect of neurostimulation on cognitive abilities.

Overall, research investigating the effect of tDCS on executive functioning and inhibitory control is mixed, particularly among those studies utilizing the Stroop task as a cognitive tool. For example, while Loftus et al. (2015) suggest anodal tDCS augments performance through an observed reduction in response times, they observe an appreciable increase in error rates following tDCS, suggesting a strategy change rather than a cognitive performance advantage. Alternatively, in a study by Frings et al. (2018), they report an increase in error rate following cathodal stimulation with no effect of anodal stimulation. However, this study failed to compare effects to a control condition and their electrode montages were different to the bihemispheric setup in this experiment. In previous studies by Fecteau et al. (2007; 2014), they fail to report on the effect of tDCS on overall response times, rendering the effect of tDCS inconclusive. Finally, a recent study by Baumert et al. (2020) reported improved response times across the various trial types of the Stroop task. However, no baseline performance was recorded and thus, one cannot say for certain that differences between stimulation groups are not resulting from inherent differences that would have existed following a baseline test prior to any intervention.

In our study, we found that when using a bihemispheric electrode montage, both anodal and cathodal stimulation (with reference to the left-DLPFC), response times and error rates (accuracy) were no different between conditions testing simple attention (i.e., control trials) or more cognitively complex inhibitory stimuli (i.e., incongruent trials). However, we did see that for males specifically, both anodal and cathodal stimulation reduced errors specifically on congruent trials compared to both control and sham conditions, with no difference in response time reductions across stimulation conditions. This finding is not explained by differences in caffeine or alcohol consumption, as all participants reported refraining from alcohol at least 24 h prior and caffeine at least 4–6 h prior to testing. Moreover, we argue that this finding is not explained by a placebo effect as 76% of participants in the sham group reported thinking they were in a neurostimulation group upon performing a manipulation check at the conclusion of the experiment.

Congruent trials are arguably the easiest trial type presented during the Stroop task, as evidenced by the fact that response times and error rates are the lowest compared to control and incongruent trial types. This is believed to result from semantic facilitation (La Heij et al., 1985; Parris et al., 2022). Moreover, it has also been established on multiple occasions that during the performance of many cognitive tasks, males often prioritize speed over accuracy, with females adopting the opposite, more cautious strategy of prioritizing accuracy (Lohman, 1986; Campbell et al., 2018; Toth and Campbell, 2019). Our findings demonstrate that during the post test (second attempt at the Stroop task), accuracy decreases for males on congruent trials as response times improve, suggesting males potentially gain confidence to adopt a strategy that prioritizes response speed on ‘easier' trials. In this case, both anodal and cathodal stimulation appear to facilitate the maintenance of accuracy performance while response times improve. The novelty of this result is noteworthy, as the aforementioned studies investigating the effect of neurostimulation on cognitive ability did not consider the effect of sex due to imbalances in participant recruitment (Loftus et al., 2015; 65% female, Frings et al., 2018; 66% female, Baumert et al., 2020; 73% female). As a result, our finding, albeit exploratory, calls into question the potential for sex to moderate the effect of neurostimulation on cognitive functioning and merits further research. Previous work has suggested differences in skull anatomy to affect the delivery of current to the central nervous system (Zamora et al., 2018; Kwan et al., 2019). However, this topic is only more recently attracting research attention as it relates to tDCS (Hunold et al., 2021; Sun et al., 2021).

When considering our N-Back results, we observed firstly that cathodal stimulation over the left dlPFC did not augment performance compared to sham or stimulation conditions. This aligns with previous work suggesting there is little evidence for cathodal stimulation to hinder working memory performance (Zaehle et al., 2011; Mylius et al., 2012; Keshvari et al., 2013). However, we did observe a trend for anodal stimulation to blunt the practice effect evident for all other conditions. This potential effect may need to be explored further or examined with increases in stimulation dosage across multiple stimulation sessions. However, recent work would suggest that repeated tDCS may not enhance the effect (Mashal and Metzuyanim-Gorelick, 2019). Overall, we did not find any significant effect of a single session of tDCS on working memory performance, a finding shared by others (Hoy et al., 2013).

Previous work has suggested that a bihemispheric bipolar montage of tDCS can be more efficacious than unipolar montages for motor tasks (Waters et al., 2017). However, we did not find any evidence of an enhanced effect from our bihemispheric montage over the dlPFC on cognitive ability. This may not be due to the electrode montage, but the stimulus waveform itself. It has been shown previously that transcranial alternating current stimulation (tACS) may be more efficacious for augmenting cognitive performance as the sinusoidal waveform can be better tuned to the underlying neural rhythms evident during cognitive processing as observed using EEG (Kim et al., 2021). Thus, further work investigating the effect of cathodal vs. anodal bihemispheric tACS on cognitive abilities is warranted.

This study set out to comprehensively investigate the effect that anodal and cathodal bipolar bihemispheric tDCS could have on executive function and working memory abilities. Overall, we provide further evidence that tDCS may not be as efficacious for enhancing cognitive functioning as it has been shown to be for motor learning. We also provide preliminary evidence that the effect of neurostimulation on cognitive functioning may be moderated by sex, with males demonstrating a benefit from both anodal and cathodal stimulation when considering performance on simple attention trial types within the Stroop task. In light of this exploratory finding, we propose a new avenue for tDCS research, that is to investigate the potential for sex to moderate the efficacy of neurostimulation on cognitive functioning.

## Data availability statement

The raw data supporting the conclusions of this article will be made available by the authors, without undue reservation.

## Ethics statement

The studies involving humans were approved by EHS research Ethics Committee, University of Limerick. The studies were conducted in accordance with the local legislation and institutional requirements. The participants provided their written informed consent to participate in this study.

## Author contributions

AT: Conceptualization, Data curation, Investigation, Methodology, Supervision, Writing – original draft. CH: Data curation, Formal analysis, Investigation, Writing – original draft. HG: Data curation, Formal analysis, Investigation, Writing – original draft. NK: Data curation, Formal analysis, Investigation, Writing – original draft. AB: Conceptualization, Methodology, Writing – review & editing. MC: Conceptualization, Formal analysis, Funding acquisition, Methodology, Resources, Supervision, Visualization, Writing – review & editing.
